# Novel *SOX10* Mutations in Waardenburg Syndrome: Functional Characterization and Genotype-Phenotype Analysis

**DOI:** 10.3389/fgene.2020.589784

**Published:** 2020-12-09

**Authors:** Supranee Thongpradit, Natini Jinawath, Asif Javed, Laran T. Jensen, Issarapa Chunsuwan, Kitiwan Rojnueangnit, Thipwimol Tim-Aroon, Krisna Lertsukprasert, Meng-Shin Shiao, Nongnuch Sirachainan, Duangrurdee Wattanasirichaigoon

**Affiliations:** ^1^Research Center, Faculty of Medicine Ramathibodi Hospital, Mahidol University, Bangkok, Thailand; ^2^Program in Translational Medicine, Faculty of Medicine Ramathibodi Hospital, Mahidol University, Bangkok, Thailand; ^3^Integrative Computational BioScience Center (ICBS), Mahidol University, Salaya, Thailand; ^4^Computational and Systems Biology Group, Genome Institute of Singapore, Agency for Science, Technology and Research, Singapore, Singapore; ^5^School of Biomedical Sciences, University of Hong Kong, Hong Kong, China; ^6^Department of Biochemistry, Faculty of Science, Mahidol University, Bangkok, Thailand; ^7^Department of Pediatrics, Faculty of Medicine, Thammasat University, Pathumthani, Thailand; ^8^Division of Medical Genetics, Department of Pediatrics, Faculty of Medicine Ramathibodi Hospital, Mahidol University, Bangkok, Thailand; ^9^Department of Communication Sciences and Disorders, Faculty of Medicine Ramathibodi Hospital, Mahidol University, Bangkok, Thailand; ^10^Division of Hematology and Oncology, Department of Pediatrics, Faculty of Medicine Ramathibodi Hospital, Mahidol University, Bangkok, Thailand

**Keywords:** *SOX10*, Waardenburg syndrome, genotype-phenotype analysis, Hirschsprung’s disease, platelet dysfunction, platelet storage pool defect

## Abstract

Waardenburg syndrome (WS) is a prevalent hearing loss syndrome, concomitant with focal skin pigmentation abnormalities, blue iris, and other abnormalities of neural crest-derived cells, including Hirschsprung’s disease. WS is clinically and genetically heterogeneous and it is classified into four major types WS type I, II, III, and IV (WS1, WS2, WS3, and WS4). WS1 and WS3 have the presence of dystopia canthorum, while WS3 also has upper limb anomalies. WS2 and WS4 do not have the dystopia canthorum, but the presence of Hirschsprung’s disease indicates WS4. There is a more severe subtype of WS4 with peripheral nerve and/or central nervous system involvement, namely peripheral demyelinating neuropathy, central dysmyelinating leukodystrophy, WS, and Hirschsprung’s disease or PCW/PCWH. We characterized the genetic defects underlying WS2, WS4, and the WS4-PCW/PCWH) using Sanger and whole-exome sequencing and cytogenomic microarray in seven patients from six unrelated families, including two with WS2 and five with WS4. We also performed multiple functional studies and analyzed genotype–phenotype correlations. The cohort included a relatively high frequency (80%) of individuals with neurological variants of WS4. Six novel *SOX10* mutations were identified, including c.89C > A (p.Ser30^∗^), c.207_8 delCG (p.Cys71Hisfs^∗^62), c.479T > C (p.Leu160Pro), c.1379 delA (p.Tyr460Leufs^∗^42), c.425G > C (p.Trp142Ser), and a 20-nucleotide insertion, c.1155_1174dupGCCCCACTATGGCTCAGCCT (p.Phe392Cysfs^∗^117). All pathogenic variants were *de novo*. The results of reporter assays, western blotting, immunofluorescence, and molecular modeling supported the deleterious effects of the identified mutations and their correlations with phenotypic severity. The prediction of genotype–phenotype correlation and functional pathology, and dominant negative effect vs. haploinsufficiency in *SOX10*-related WS were influenced not only by site (first two vs. last coding exons) and type of mutation (missense vs. truncation/frameshift), but also by the protein expression level, molecular weight, and amino acid content of the altered protein. This *in vitro* analysis of *SOX10* mutations thus provides a deeper understanding of the mechanisms resulting in specific WS subtypes and allows better prediction of the phenotypic manifestations, though it may not be always applicable to *in vivo* findings without further investigations.

## Introduction

Waardenburg syndrome (WS) is a single-gene disorder characterized by congenital onset sensorineural hearing loss, focal skin pigmentation abnormality (white forelock and focal depigmented skin), blue iris with or without heterochromia, and other abnormalities of neural crest-derived cells (Nayak and Isaacson, 2003; Van Camp and Smith, 2019). WS can be further classified based on additional features: the presence of dystopia canthorum (lateral displacement of the inner canthi of the eyes) distinguishes WS type I (WS1; OMIM 193500) and WS type III (WS3; OMIM 148820) from WS type II (WS2; OMIM 193510) (Liu et al., 1995; Van Camp and Smith, 2019); WS3, or Klein–Waardenburg syndrome, is a more severe form of WS1 and is usually associated with musculoskeletal abnormalities of the upper extremities; and WS type IV (WS4), or Waardenburg–Shah syndrome, is characterized by the distinctive combination of WS2 and Hirschsprung’s disease, chronic constipation, or pseudo-gut obstruction (Liu et al., 1995; Van Camp and Smith, 2019). Some patients with WS4 may present with neurological features including peripheral demyelinating neuropathy and/or central dysmyelinating leukodystrophy, leading to a syndrome called PCW or PCWH (peripheral demyelinating neuropathy, central dysmyelinating leukodystrophy, Waardenburg syndrome, and Hirschsprung’s disease) (Pingault et al., 1998, 2000; Toki et al., 2003; Van Camp and Smith, 2019). The peripheral neuropathy is clinically characterized by muscle wasting/atrophy, hyporeflexia and autonomic dysfunction, which can be objectively documented by abnormal nerve conduction velocity and depleted myelination shown by the pathological examination of nerve biopsy (Inoue et al., 2004). The central nervous system involvement can be demonstrated by reduced myelination shown on brain magnetic resonance imaging (MRI) and neuropathological study, in addition to clinical findings of hypotonia, spastic diplegia, ataxia and nystagmus, global developmental delay, and autism (Inoue et al., 2004).

The most prevalent subtype of WS is WS1, followed by WS2, WS4, and WS3. WS1 and WS3 are caused by mutations in *PAX3* and rarely *EDNRB* gene. WS2 are associated with mutations in *MITF* (15%), *SOX10* (16%) and less common *SNAI2* gene (Bondurand et al., 2007; Chaoui et al., 2011; Baral et al., 2012; Zhang et al., 2012; Song et al., 2016; Li et al., 2019; Van Camp and Smith, 2019). The most common causes of WS4 (WS4C, OMIM 613266) and PCW/PCWH (OMIM 609136) are mutations in *SOX10*, which accounts for 45%–55% of cases, followed by pathogenic variants of *EDN3* and *EDNRB* in 20%–30%, and lastly *SNAI2* mutations (Puffenberger et al., 1994; Pingault et al., 1998; Bondurand et al., 2007; Chaoui et al., 2011; Zhang et al., 2012; Song et al., 2016; Li et al., 2019; Van Camp and Smith, 2019). WS4 is thus the most genetically heterogeneous form of WS.

The aim of this study was to determine the genetic defects underlying WS2 and WS4, and to analyze genotype–phenotype correlations using *in vitro* studies and molecular data.

## Materials and Methods

### Research Subjects

Patients with a clinical diagnosis of WS2, WS4, or PCW/PCWH who were followed up at Ramathibodi Hospital and Thammasat University Hospital were invited to participate in the study. Medical records and investigations including temporal bone computed tomography and brain magnetic resonance imaging were reviewed. The diagnosis of Hirschsprung’s disease was confirmed by radiological and/or pathological evidence. The diagnosis of PCW/PCWH was made primarily based on concomitant symptoms of hypotonia, developmental delay, and its related findings with or without brain MRI and neuropathological studies.

### Sanger Sequencing of *MITF* and *SOX10* Genes

Genomic DNA was extracted from peripheral blood of the participants using Purgene DNA extraction kit. Because *MITF* and *SOX10* mutations are recognized as the leading causes of WS2 and WS4, we initially performed polymerase chain reaction–Sanger sequencing of the entire coding sequences of these two genes as initial genetic screening, and further analyzed *EDN3*, *EDNRB*, and *SNAI2* genes in the event of negative results for *MITF* and *SOX10*. Primers were designed using PRIMER3^1^ and the primer sequences are provided in Supplementary Tables 1,2. Evidence for the pathogenicity of the variants was obtained by bioinformatics analysis using SIFT^2^, PolyPhen2^3^ and splice site prediction by neural network^4^ mutation/variant databases, including ClinVar^5^ and the Human Genome Mutation Database (HGMD)^6^; population database including the gnomAD allele frequencies; and CADD scores. Interpretation of the variants was based on the guideline of American College of Medical Genetics and Genomics (ACMG) and the Association for Molecular Pathology (AMP) (Richards et al., 2015), using the web-based analysis tool, VARSOME^7^.

### Cytogenomic Microarray (CMA) and Whole-Exome Sequencing (WES)

Cytogenomic microarray and trio/quartet WES were performed in patients with additional or unusual phenotypes and/or negative results following *MITF* and *SOX10* gene Sanger sequencing. The WES performed was to detect mutations in *EDN3*, *EDNRB*, and *SNAI2*, in addition to *SOX10*. Moreover, the WES analysis could detect other monogenic disorder associated with the unusual phenotypes including developmental delay and platelet disorders of our patients. The CMA was to identify coincidental chromosomal disorder which could explain the unusual phenotypes and psychomotor delay.

Cytogenomic microarray was performed using single nucleotide polymorphism array (Illumina Infinium CytoSNP-850K BeadChip) and analyzed using BlueFuse Multi software v4.1. The Database of Genomic Variants and the Thai CNV database were used to exclude common structural variations (Suktitipat et al., 2014).

Whole-exome sequencing was performed by Macrogen, South Korea (Illumina HiSeq2500), using SureSelect (V5+UTR; Agilent) for target capture (100 bp Pair End mode and 125x coverage). The quality of the data was assessed by FastQC and the data was processed following Broad Institute’s best practice guidelines for GATK v3.4^8^ and previous established methods (Van der Auwera et al., 2013).

Standardized human phenotype ontology (HPO) terms were used to describe the phenotypes as follows: HPO:0008527, congenital sensorineural hearing impairment; 0011869 abnormal platelet function; 0001022, albinism; 0001107, ocular albinism; 0007443, partial albinism; and 0001249, intellectual disability. In addition, a *de novo* heterozygous for autosomal dominant genes, hemizygous for X-linked genes, and homozygous or compound heterozygous for autosomal recessive genes were also analyzed for candidates which may explain the unusual phenotypes. Each variant was subjected to Sanger sequencing to confirm its presence in the proband and familial samples.

### Plasmids, Cell Culture, Transfection, and Reporter Assay

The *SOX10* expression plasmid (pCMV-3xFlag-SOX10) and luciferase reporter containing the human *MITF* promoter (pGL3-MITF-Luc or pCMV β-galactosidase) were kindly provided by Prof. Yong Feng and Prof. Jia-Da Li (Central South University, Hunan, China). Site-directed mutagenesis was carried out using a QuikChange II XL Site-Directed Mutagenesis Kit (Agilent, United States), according to the manufacturer’s instructions, using the mutagenesis primers shown in Supplementary Table 3. The plasmid constructs were selected by diagnostic restriction digestion (*Xba*I and *Eco*RI) and confirmed by sequencing before transient transfection for luciferase and beta-galactosidase activity assays, as described previously (Zhang et al., 2012).

HEK293T (human embryonic kidney) or NIH/3T3 (mouse embryonic fibroblast) cells (ATCC) were cultivated in Dulbecco’s modified Eagle’s medium complemented with 10% fetal bovine serum, 100 U/ml penicillin, and 100 μg/ml streptomycin, under 5% CO_2_ at 37°C for 24–48 h prior to transfection. Cultured cells at 80–90% confluence were transfected with the transfection mix, using a ratio of 3 μl Lipofectamine 2000 (Invitrogen Corporation, Carlsbad, CA, United States) per 1 μg of total DNA transfected, according to the manufacturer’s protocol. pCMV10 3X Flag and pCMV β-galactosidase were used for normalization and monitoring the transfection efficiency, respectively.

At 24 h after transfection, lysates were prepared from the cultured HEK293T cells, using 1 × lysis reagent (Promega, United States) and then assayed for luciferase and β-galactosidase activities using an Infinite 200 PRO plate reader (Tecan, Switzerland). The reporter assays were carried out three times in triplicate, on different days, using various batches of cells.

### Western Blot Analysis

SOX10 protein levels in transfected HEK293T cells were verified by western blot analysis. Cultured cells were harvested 24 h post-transfection and protein was isolated using RIPA lysis buffer (Bio-Rad), according to the manufacturer’s instructions. Proteins (4 μg) were subjected to 12% sodium dodecyl sulfate-polyacrylamide gel electrophoresis and transferred to a polyvinylidene fluoride membrane. The membrane was blocked using blocking solution (3% bovine serum albumin in TBS plus 0.1% Tween-20) for 2 h and then stained with mouse monoclonal anti-Flag M2 antibody (1:1000 dilution; Sigma, St Louis, WA, United States) and goat anti-mouse glyceraldehyde 3-phosphate dehydrogenase (GAPDH) antibody (Thermo Scientific) as a protein-loading control. After washing four times with washing buffer (TBS plus 0.1% Tween-20) for 15 min each, the membrane was stained for 1 h with goat anti-mouse IgG secondary antibody tagged with horseradish peroxidase (1:1000 dilution; Thermo Fisher Scientific) at room temperature, followed by four washes.

Protein expression levels were quantified using Luminata Forte Western HRP substrate (Merck, United States) and the ChemiDoc MP Imaging System (Life Science Research). Relative quantities of proteins visualized on the blot were measured using Image Lab (ver. 6.0.1; Bio-Rad Laboratories, Inc.). The amount of each SOX10 protein was measured compared to the wildtype, which was set as 1, and the amount of the GAPDH in each lane was determined and compared to the GAPDH in the wild-type lane, which was set as 1. The amount of each SOX10 variant was then calculated relative to the amount of GAPDH in each individual lane, using the arithmetic rule of three (Supplementary Figure 1).

### Immunofluorescence Study

NIH3T3 cells were grown on 6-well plates with cover-slides and transfected with pCMV-3xFlag-*SOX10* (wild-type *SOX10*) or *SOX10* variant expression plasmids, following an established protocol (Zhang et al., 2012). At 48 h after transfection, the cells were washed with phosphate-buffered saline (PBS), fixed with 4% paraformaldehyde at room temperature for 30 min, and permeabilized in PBS plus 0.2% Triton X-100 (Scharlau, Spain) for 1 h. The reaction was then stopped with blocking solution (PBS, 3% bovine serum albumin plus 5% goat serum) at room temperature for 1 h, and the slides were stained with mouse monoclonal anti-Flag M2 primary antibody (1:600 dilution; Sigma) at 4°C overnight, washed three times with PBS plus 0.1% Triton X-100, and then incubated for 2 h with DyLight 488 fluorescence-labeled secondary goat anti-mouse antibody (1:300 dilution; Thermo Fisher Scientific). The cells were then incubated with 4′,6′-diamino-2-phenylindole (DAPI, Invitrogen) for 3 min before immunofluorescence analysis using a laser scanning confocal microscope (Nikon, Japan) and the NIS-Elements Viewer software.

### Molecular Modeling

The structure of full length SOX10 as well as frame shift mutants, Phe392Cysfs^∗^117 and Tyr460Leufs^∗^42 were predicted using I-TASSER (Iterative Threading ASSEmbly Refinement) (Zhang, 2008; Roy et al., 2010; Yang et al., 2015). Template structures utilized to model SOX10 included *Cylindrotheca fusiformis* pleuralin-1; 2NBI (De Sanctis et al., 2016); HMG-box domain of human SRY, 1J46 (Murphy et al., 2001); complex containing transcription elongation factor SPT6, 6gmh (Vos et al., 2018); HMG box of human SOX-17, 2yul (Abe et al., 2011); chimera of transcription factor SRY and high-mobility group protein HMGB1, 2gzk (Stott et al., 2006); Human SOX-9 HMG domain bound to DNA, 4euw (Joint Center for Structural Genomics [JCSG], 2019); and mouse SOX17 bound to DNA, 3F27 (Palasingam et al., 2009). The structure of the isolated high mobility group (HMG) domain was modeled with Swiss-Model using the structure of SOX17 bound to DNA (3F27) (Palasingam et al., 2009) as a template. The DNA chain in the models generated by I-TASSER or Swiss-Model was incomplete or absent. The DNA structure from SOX17 bound to DNA (3F27) was added by aligning the HMG domain in the SOX10 model and SOX17 structure. Structures were viewed using the PyMOL Molecular Graphics System, version 2.0.6 (Schrödinger, LLC, NY, United States).

## Results

A total of seven patients were enrolled in the study, including two with WS2 and five with WS4 (1 classic WS4 and 4 WS4-PCW/PCWH). Two of the 4 WS4-PCW/PCWH patients were twin siblings, they were naturally conceived and believed to be monozygotic twins based on the history of monochorion and the sharing of similar facial and clinical phenotypes including platelet disorders and the same *de novo* SOX10 pathogenic allele. Forensic study was not performed to confirm the monozygosity. The ages of the patients at study entry were ranging 4–13 years. All the patients had no family history of hearing loss, and no dystopia canthorum, white forelock, or anosmia/hyposmia. Four patients had developmental delay, hypotonia, and/or autism. Unusual manifestations were detected in four patients, including platelet dysfunction in two siblings, ptosis in one, and hypogonadism in the other (Table 1).

**TABLE 1:** Clinical manifestations and genotypes including proposed pathologic mechanisms associated with *SOX10* mutations.

**Family**	**Pt**	**Exon: nucleotide change**	**Protein effect**	**WS subtype**	**Mechanism**	**Clinical findings**				**ClinVar accession number**
						**Blue iris**	**Intestinal**	**Neuro**	**Others**	
WS-01	1	Ex4: c.89C > A	p.Ser30*	WS4	Haplo	+	constipation^*b*^	no	no	SCV001245531
WS-02	2A	Ex5: c.1155_1174dup GCCCCACTATGG CTCAGCCT	p.Phe392Cysfs*117	WS4-PCW	Toxic/Gain-of-function	+	no	infantile hypotonia, GDD	bleeding disorder	SCV001245527
	2B					+	no	infantile hypotonia, GDD, ADHD	bleeding disorder	
WS-03	3	Ex3: c.425G > C	p.Trp142Ser	WS4-PCWH	DN, potent	+^*a*^	constipation^*b*^	infantile hypotonia, GDD	ptosis of left eye	SCV001245534
WS-04	4	Ex5: c.1379delA	p.Tyr460Leufs*42	WS2	DN, weak	+	no	no	no	SCV001245532
WS-05	5	Ex3: c.207_8 delCG	p.Cys71Hisfs*62	WS4-PCWH	DN, potent	+	constipation^*b*^	GDD	no^*c*^	SCV001245533
WS-06	6	Ex3: c.479T > C	p.Leu160Pro	WS2	DN, weak/Haplo	+	no	no	hypogona dism; abnormal CT^*d*^	SCV001245525

*^*a*^with segmental heterochromia of both eyes; ^*b*^chronic constipation but radiologic investigation did not confirm Hirschsprung’s disease; ^*c*^with normal temporal bone CT scan; ^*d*^hypogonadotropic hypogonadism, micropenis, right undescended testis; temporal bone CT scan showing dilatation of bilateral vestibules and semicircular canals, normal 2 ½ turned cochlear and vestibular aqueduct. ADHD, attention deficit and hyperactivity disorder; DN, dominant negative mechanism; GDD, global developmental delay**;** Haplo, haploinsufficiency, Pt, patient; 2A and 2B representing twin A and twin B of family 2, respectively.*

### Identification of Six Novel *SOX10* Mutations

Conventional sequencing revealed no *MITF* mutations but six distinct heterozygous *SOX10* variants. CMA and the trio/quartet WES were performed in three probands with atypical phenotypes, including bleeding disorders (2 patients) and ptosis (1 patient). CMA failed to detect any chromosomal abnormalities. The WES confirmed the *SOX10* mutations identified by conventional sequencing in the three individuals, and revealed no other pathogenic/candidate variants. These finding likely excluded alternative genetic disorder which may explain the unusual phenotypes.

**FIGURE 1:**
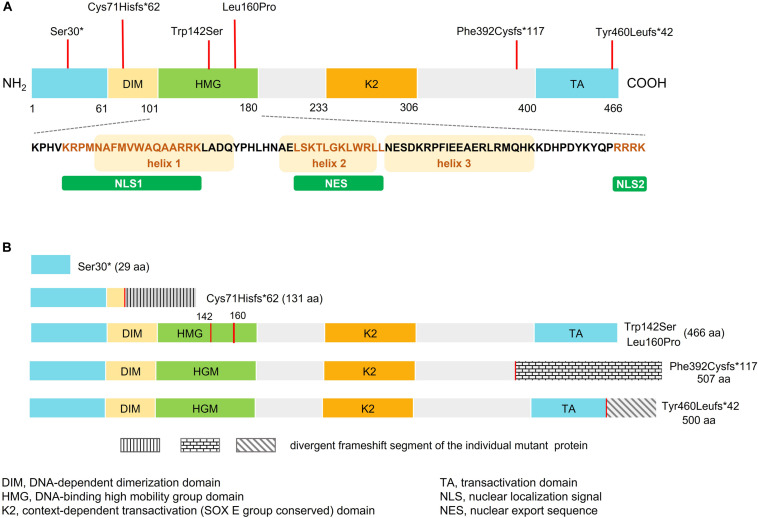
Schematic representation of wild-type and mutant SOX10. **(A)** Domain structure of SOX10 showing dimerized domain (DIM), high mobility group domain (HMG), and C-terminal transactivation (TA) domain. Bottom panel shows amino acid sequence of the HMG domain, three alpha-helical domains, two NLSs (NLS1 and NLS2), and the nuclear export sequence (NES). The orange box represents the helix fragment while the different colored text represents sequence of the NLS1, NES, and NLS2. Location of the SOX10 mutations identified in this study are indicated by red vertical lines above the domain structure. **(B)** Predicted domain structure of each mutant SOX10.

**FIGURE 2:**
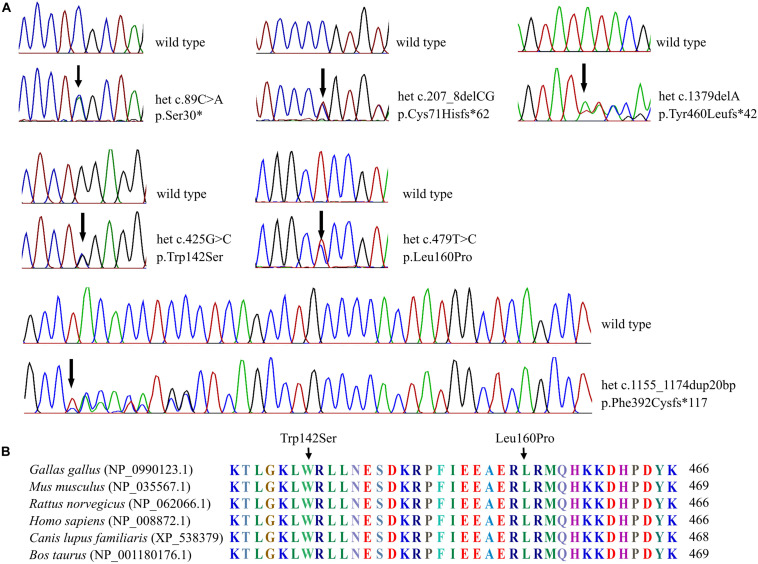
Sequenograms and protein alignment. **(A)** Sequenograms of the six *SOX10* mutations. **(B)** Protein sequence alignment of vertebrate SOX10; note the highly conserved Trp142 and Leu160 residues across various species.

The six *SOX10* mutations identified in this study were: c.89C > A (p.Ser30^∗^) and c.207_8 delCG (p.Cys71Hisfs^∗^62) in exon 3; c.479T > C (p.Leu160Pro) in exon 4; and c.1379 delA (p.Tyr460Leufs^∗^42) in exon 5; c.425G > C (p.Trp142Ser) in exon 3; and a 20-nucleotide insertion, c.1155_1174dupGCCCCACTATGGCTCAGCCT (p.Phe392Cysfs^∗^117), in exon 5. The location and schematic representation of each variant of the mutant are shown in Table 1 and Figure 1. These variants did not affect the last base of exons or exon-intron boundaries, therefore unlikely to cause splicing error. All the mutations identified in this study were novel *de novo* mutations not found in the parents (Figure 2A). Four variants were classified as pathogenic based on basic criteria of ACMG/AMP guidelines and/or CADD score calculation, as follow: Ser30^∗^ (PVS1, PM2, PP3, PP5; CADD score 35, damaging; p.Cys71Hisfs^∗^62 (PVS1, PM2, PP3); p.Phe392Cysfs^∗^117 (PVS1, PM2, PP3, PP5) and p.Tyr460Leufs^∗^42 (PVS1, PM2, PP3, PP5). The two missense variants were classified to be likely pathogenic as follow: p.Trp142Ser (PS2, PM1, PM2, PM5, PP2, PP3, PP5; CADD score 28.2, damaging) and p.Leu160Pro (PS2, PM1, PM2, PP2, PP3, PP5; CADD score 28.9, damaging). All these variants were not present in gnomAD (allele frequency = 0). The Trp142 and Leu160 residues were highly conserved across SOX10 in various species of vertebrates (Figure 2B), indicating the functional importance of these two amino acids (aas). The reference sequences of the *SOX10* studies were: NT_011520.11, NM_006941.3, NP_008872.1.

### *MITF* Promoter Transactivation, Western Blot and Subcellular Localization

**FIGURE 3:**
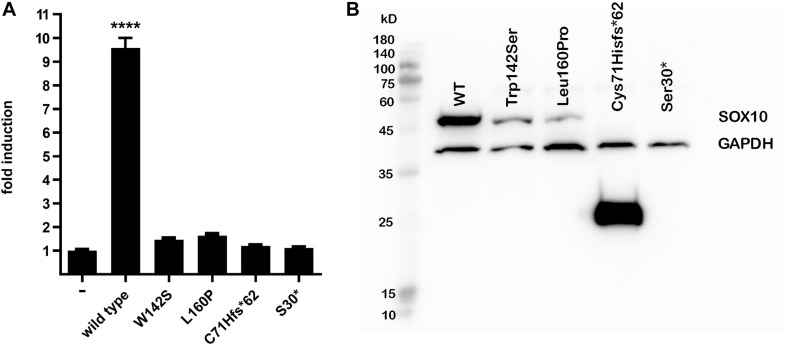
*MITF* transactivation activity and western blot. **(A)** *MITF* transactivation (TA) activity. Note 9.5-fold induction of TA activity by wild-type SOX10 compared with empty vector; nearly complete loss (2%) of TA activity of Cys71Hisfs*62 and Ser30*, and 1.5-fold and 1.7-fold induction by Trp142Ser and Leu160Pro compared with empty vector (i.e., 6 and 8% of wild-type control after deduction of activity of empty vector, respectively). Results shown are representative of at least three independent experiments (****p < 0.001 by one-way ANOVA). **(B)** Western blot analysis. From left to right: molecular size marker, wild-type SOX10, Trp142Ser and Leu160Pro variants of normal predicted size at 51 kD, Cys71Hisfs*62 25 kD, and undetectable Ser30* at 3.1 kD. Note marked reduction in proteins compared with wild-type for Trp142Ser (30% residual protein) and Leu160Pro (11% residual amount) and increase for Cys71Hisfs*62 variant (500%). GAPDH was used as a loading control for normalization for wild-type SOX10 and each variant. Note similar GAPDH levels in all lanes. Image brightness and contrast not modified from original. Size marker: PM2510, ExcelBand, SMOBIO.

SOX10 transactivates the *MITF* promoter. We therefore determined if the SOX10 variants lost the ability to transactivate this downstream gene. The p.Ser30^∗^ and p.Cys71Hisfs^∗^62 mutations were associated with almost complete loss of transactivation activity on the *MITF* promoter, while the activities of the p.Trp142Ser and p.Leu160Pro variants were significantly reduced compared with the wild-type (Figure 3A). Western blot analysis revealed that p.Trp142Ser and p.Leu160Pro were also associated with significantly reduced SOX10 protein synthesis, undetectable synthesis of the p.Ser30^∗^ protein, and excessive production of the p.Cys71His^∗^62 protein variant (Figure 3B). *In vitro* functional analysis of the elongation mutations p.Tyr460Leufs^∗^42 and p.Phe392Cysfs^∗^117 was attempted but was unsuccessful because of technical difficulties.

**FIGURE 4:**
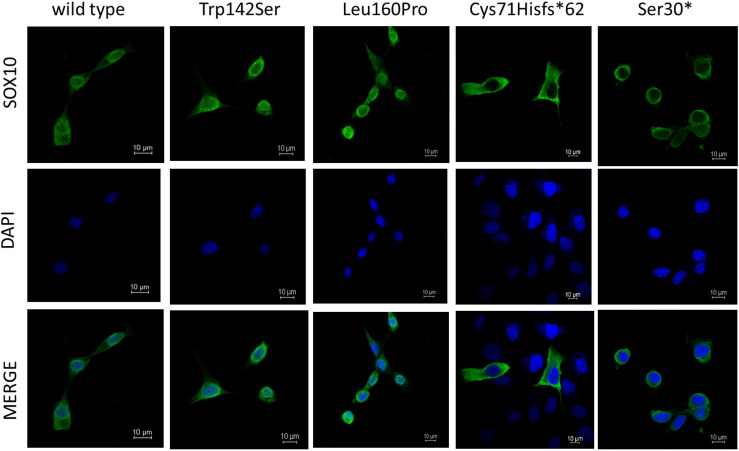
Subcellular localization of SOX10 variants. Wild-type and mutant SOX10 proteins shown in green and DAPI, revealing nucleus, shown in blue.

As for Immunofluorescence analysis, transfected cells expressing GFP for each SOX10 variant construct were examined for the subcellular localization of GFP-fused SOX10 protein, then classified into three categories as follows: nuclear, cytosolic, and nucleocytosolic. The majority of the transfected cell with wild-type, p.Trp142Ser, and p.Leu160Pro variants, showed nuclear localization at 82, 83, and 85%, in respective order (Supplementary Table 4 and Figure 4). Concerning the two truncated variants, p.Cys71Hisfs^∗^62 and p.S30^∗^, the SOX10 proteins were mainly expressed in cytosolic compartment at 100 and 93% of the cells studied, respectively.

### Molecular Models of SOX10 Protein Variants

**FIGURE 5:**
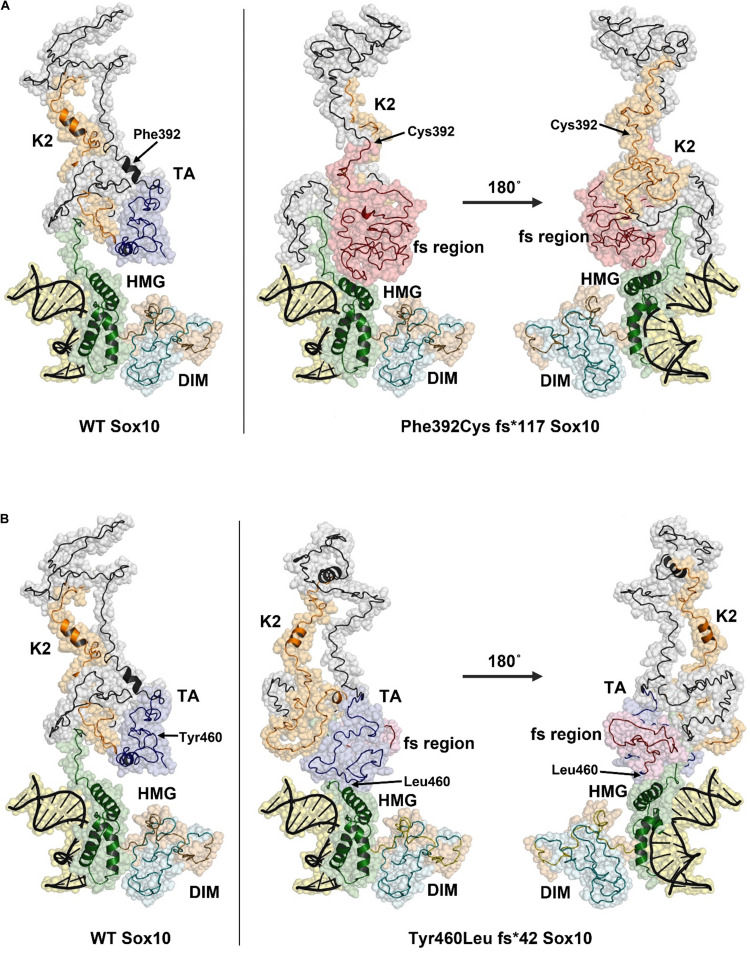
Structural models of Phe392Cysfs*117 and Tyr460Leufs*42 SOX10 frame-shift (fs) variants. The position of Phe392 and Tyr460 are indicated with arrows in the WT structure model (left panel). The positions of substituted residues are indicated with arrows in the mutant structures. Amino acid residues that result from the frame shift mutation are shown in red. **(A)** Model of Phe392Cys fs*117 Sox10, the HMG domain is compacted due to the presence of the fs region and may impact DNA binding. **(B)** The Tyr460Leu fs*42 model predicts a change in the TA structure with additional contacts between the TA/fs region and the HMG domain. This structural change may reduce activity of the TA region.

The predicted structure of the full-length SOX10 protein constructed using I-TASSER using template-based fragment assembly simulations contained a highly ordered HMG region with a three-helix bundle. This domain was highly similar to the structure of SOX9 and other SOXE proteins. Outside the HMG domain, SOX10 was predicted to be disordered with only a few short helical regions. The C-score for the SOX10 model, as an estimate of model quality, was −3.21, indicating low confidence in regions of the predicted structure, possibly as a consequence of the disordered nature of the protein. The C-terminal region of the SOX10 protein was predicted to be stabilized through interactions between the K2 and transactivation (TA) regions (Figure 5A, left panel).

The model for the p.Phe392Cysfs^∗^117 SOX10 mutation indicates a substantial structural change occurs due to the extended amino acid sequence in this variant. The frame shift region, shown in red, causes new contacts with the HMG domain, pushing one of the three helices into a more compact configuration (Figure 5A, right panel). The K2 region of p.Phe392Cysfs^∗^117 SOX10 is highly disordered and the two short helices seen in the wild type protein are absent. The structures of the K2 regions are distinct between the wild type and p.Phe392Cysfs^∗^117 SOX10. K2 from p.Phe392Cysfs^∗^117 SOX10 is less compact and predicted to make contacts with the HMG domain. The TA region is not present due to the amino acid sequence change from the frameshift mutation. The structural changes in p.Phe392Cysfs^∗^117 SOX10 may have several effects including altering DNA binding affinity, limiting transactivation activity, and reducing the overall stability of the protein.

The p.Tyr460Leufs^∗^42 mutation would extend the TA region, eliminating six residues of the SOX10 protein. The predicted structural changes due to the p.Tyr460Leufs^∗^42 mutation are less substantial compared to the Phe392Cysfs^∗^117 variant (Figure 5B). The HMG domain is essentially unchanged compared to the WT SOX10 structure model. However, the folding of the K2 and TA regions is altered in the p.Tyr460Leufs^∗^42 mutant. The well-ordered contact seen in the WT model between the K2 and TA regions is absent in the p.Tyr460Leufs^∗^42 mutant. In addition, new contacts are predicted between the TA/fs region and the HMG domain. It is likely that these changes in the p.Tyr460Leufs^∗^42 would negatively impact the activity of the transactivation domain. An effect on protein stability for p.Tyr460Leufs^∗^42 is possible due to the increased disorder in the K2/TA regions.

The frame-shift and truncation mutations p.S30^∗^ and p.Cys71Hisfs^∗^62 were not modeled, however, the effect of these variants can be inferred by their location in the SOX10 structure model. The p.S30^∗^ and p.Cys71Hisfs^∗^62 mutations do not contain the majority of the SOX10 protein including the HMG domain (DNA binding region) and predicted nuclear localization signals (NLSs). These mutants are therefore unlikely to form a homodimer or interact with other SOX10 proteins, given that the HMG domain has been reported to be important for dimer formation (Ramsook et al., 2016).

**FIGURE 6:**
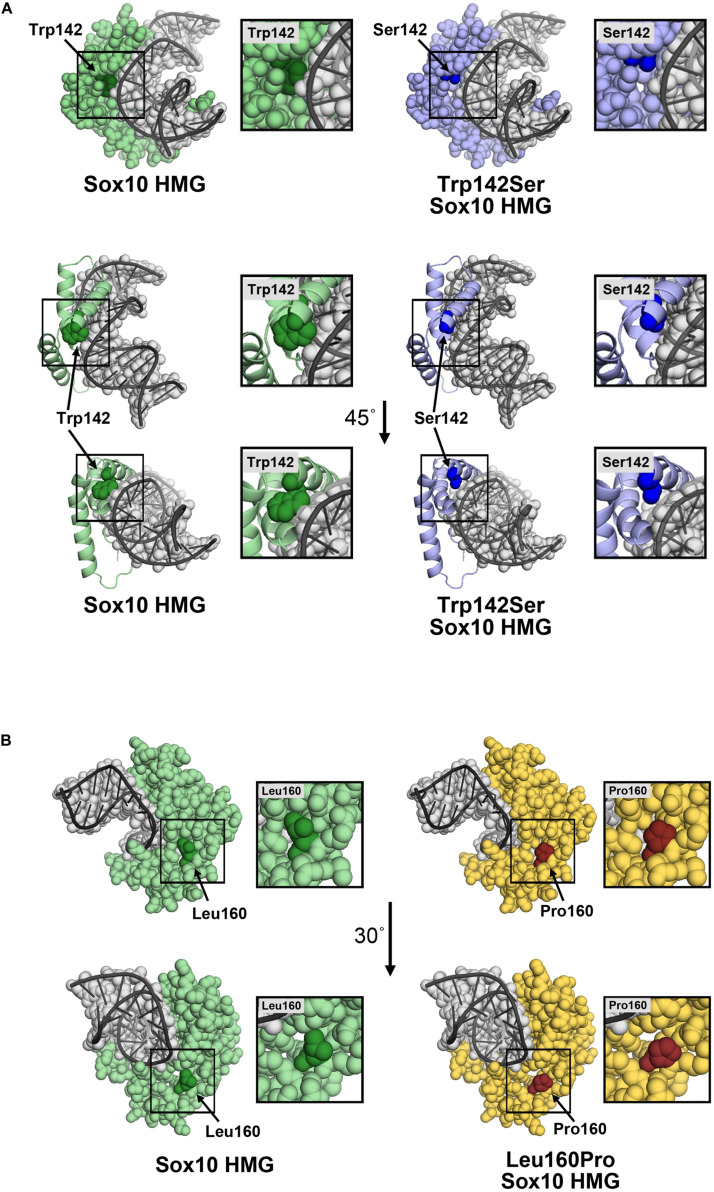
Molecular models of p.Trp142Ser and p.Leu160Pro. **(A)** Model of the SOX10 HMG domain showing Trp142 and Ser142 substitution. The SOX10 HMG domain generated using Swiss-Model is presented as a space-filling model (upper panel) or ribbon diagram (lower panel) with only residue 142 with space-filling representation. Wild-type Trp142 is shown in dark green and other residues in light green. The Trp142Ser substitution is shown in dark blue and other residues in light blue. The DNA strand (gray) is shown as an overlay of the space-filling and ribbon formats. Contacts between Trp142 and DNA were noted. Substitution of Trp142 with Ser eliminated contact between residue 142 and the DNA chain. **(B)** Model of SOX10 HMG domain showing Leu160 and the Pro160 substitutions. The SOX10 HMG domain generated using Swiss-Model is presented as a space-filling model. Wild-type Leu160 is shown in dark green and other residues in light green (left panel). The Leu160Pro substitution is shown in red and other residues in yellow (right panel). Residue 160 does not make direct contact with the DNA chain.

Substitution of Trp with Ser at position 142 leaves an open region in the HMG domain. The model does not predict a specific structural change, however, the p.Trp142Ser substitution would be expected to increase the flexibility of the HMG domain, and perturbations in the structure may affect its association with DNA. Possible contacts between Trp142 and DNA were absent in the p.Trp142Ser variant (Figure 6A). Therefore, this loss of contact could aggravate the deleterious effect of the variant, in addition to the reduced protein synthesis and stability. In addition, Tryptophan is a non-polar hydrophobic amino acid with aromatic side chain, it often plays a role in binding to non-protein atoms including DNA; therefore replacement by serine, a polar amino acid, could likely be disastrous (Betts and Russell, 2003). Substitution of Leu160 with Pro was not predicted to significantly change the structure of the HMG region (Figure 6B), although Pro at 160 is less bulky and may produce a gap in the structure leading to increased flexibility, and possibly altered stability/interactions. While residue 160 is not in proximity to DNA, changes in DNA affinity of the p.Leu160Pro variant cannot be ruled out.

### Genotype–Phenotype Correlation

The truncated mutations, p.Ser30^∗^ and p.Cys71His^∗^62, were found in patients with classic WS4 and PCWH, in orderly. The p.Trp142Ser and p.Leu160Pro, were associated with PCWH and WS2, respectively. As for the elongation mutations, the p.Tyr460Leufs^∗^42 linked with WS2 phenotype, whereas the p.Phe392Cysfs^∗^117 was present in patients with PCW with bleeding diathesis.

Given the lack of HMG and TA domains, p.Ser30^∗^ failed to transactivate the *MITF* promoter. The pre-mature mRNA would be subjected to nonsense-mediated mRNA decay (NMD), leading to significantly reduced protein synthesis. However, a mutant protein was also produced, as shown by immunofluorescence study, but the protein was too small (3.1 kD) to be detected by western blot. Despite its lack of a NLS, the p.Ser30^∗^ variant was partly present in the nucleus, probably as a result of passive diffusion of the small (<63 kD) protein through the nuclear pore complex (Gorlich and Mattaj, 1996; Junod et al., 2020). In summary, the molecular evidence supports a haploinsufficiency mechanism for this variant, resulting in classic WS4.

The detected size of the p.Cys71Hisfs^∗^62 variant (25 kD) was larger than that estimated based on the aa residues (13.6 kD), possibly because of post-translational modification. The altered protein lost its nuclear localization and transactivation properties (Figures 3A, 4), consistent with its lack of an NLS region and TA domain (Figure 1B). The p.Cys71Hisfs^∗^62 variant was assumed to enter the nuclear compartment by passive diffusion, but this was not observed. In addition to molecular weight, an aberrant charged/hydrophobic aa ratio is also known to affect the nuclear transport of proteins, which might explain the restriction of this mutant protein to the cytosol (Junod et al., 2020). Surprisingly, the *in vitro* data demonstrated high protein expression levels of this variant, indicating unactivated NMD machinery, despite the fact that the mutation was located in the first coding exon (exon 3) of the *SOX10* gene. The high levels of this protein likely led to a strong dominant-negative effect of the variant, resulting in a severe phenotype (PCWH), as noted in this case. Without these data from the *in vitro* analysis, the variant might have been predicted to cause a milder WS2 or classic WS4 phenotype.

The p.Trp142Ser and the p.Leu160Pro mutations could interfere with helix formation (helix 2 and 3) in SOX10, with severe effects on DNA-binding and prohibiting *MITF* transactivation, despite having an intact TA domain. These missense variants were expected to induce dominant-negative mechanisms, producing a severe phenotype. Unexpectedly, protein expression levels of the p.Trp142Ser variant were reduced to 30% and levels of the p.Leu160Pro variant to 11%, compared with the wild-type (Figure 3B and Supplementary Figure 1). However, only the p.Trp142Ser mutation was associated with the most severe phenotype, PCWH, while the p.Leu160Pro mutation caused WS2. This discrepancy can be explained by the differential expression of the missense proteins, with higher levels of the mutant protein causing more severe damage. Trp142 is one of the four aas (Trp 114 and 142; Phe 111 and 153) forming the hydrophobic core of the HMG domain at the intersection of the three helices. Although p.Trp142Ser did not cause fundamental structural changes, as displayed by the molecular model, the potentially unstable contact between the HMG domain and the bound DNA could result in defective DNA binding. Together with the lower abundance of protein, the p.Trp142Ser led to dominant negative mechanism. The milder phenotype associated with p.Leu160Pro suggests a weak dominant-negative mechanism or potential haploinsufficiency associated with the marked decrease of protein produced.

Functional pathology and prediction of clinical severity of the extension mutations is more complicate. There are at least two examples of an in-frame SOX10 variants consisting normal SOX10 protein with additional (82 and 86) amino acids attached to its carboxyl terminus, creating a mutant fusion protein leading to PCWH phenotypes (Sham et al., 2001; Inoue et al., 2007). An extended functional study including competition assay, by Inoue et al., has demonstrated that the specific extension variant (c.1400del12bp) with 82 amino acids tail showed no dominant negative interference with the wildtype but a unique sequence of 11 amino acids enriched with tryptophan and arginine (WR domain) contained in the extension tail was responsible for the pathologic properties by toxic and gain-of-function mechanism (Inoue et al., 2007). As for out-of-frame mutation, its functional pathology may be different. Clinical severity of these frameshift variants likely depends on the location of the premature truncation, the more proximal it is the more severe the phenotype (Inoue et al., 2007).

Based on the aforementioned basis, the p.Tyr460Leufs^∗^42, the frameshift variants identified in our study is likely to result in the less severe phenotype which is corresponding to the WS2, as observed in patient 4. On the other hand, the p.Tyr460Leufs^∗^42, located in the last coding exon of *SOX10*, is predicted to not activate the NMD machinery but to have a dominant-negative effect leading to severe phenotype. Plausible explanations for the less severe manifestation than expected, include the intact of TA domain, K2–TA interaction and transactivation activities, as shown in the structural model, and that the extension tail does not majorly disrupt the conformation of the protein, in sum leading to a weak dominant-negative allele.

**FIGURE 7:**
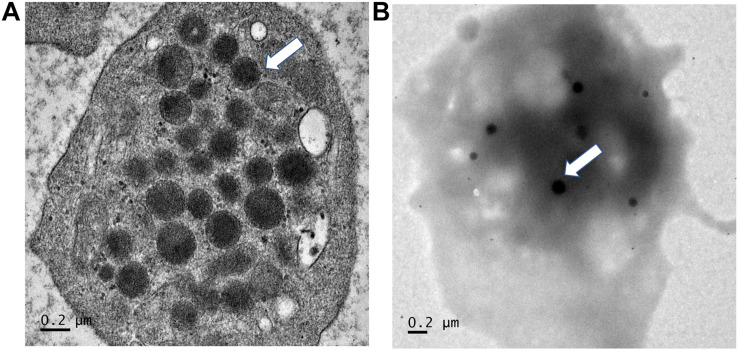
Electron microscopy findings of platelets. Platelet transmission electron microscopy (Phillips 301 electron microscope; F.E.I., Hillsboro, OR, United States) study of patients 2 and 3, using whole-mount method (White, J. G., 2008). **(A)** Note normal numbers of alpha granules (normal range 30–50 per platelet). **(B)** Normal number of dense granules (in 30 healthy subjects, 2.4–3.7 per platelet). Magnification ×25, 200.

Regarding p, Phe392Cysfs^∗^117, it eliminates the entire TA domain which is likely to result in a complete loss of transactivation activity and the most severe phenotype. Also, the variant includes a long sequence thread of unrelated 116 amino acids. Given the marked change of the protein primary structure and the additional phenotype (platelet dysfunction) in this case, we speculate that toxic or gain-of-function is likely to be the pathologic mechanism. However, without competition assay, the dominant negative mechanism cannot be completely excluded. The bleeding disorder in the patients with p.Phe392Cysfs^∗^117 variant was first noted at age around 3–4 years, when frequent epistaxis causing iron-deficiency anemia was evident. Investigations showed normal platelet counts of 320,000–385,000/mm^3^ and normal platelet morphology under the microscope, but prolonged bleeding times (12–15 min; normal ≤ 10 min) and normal activated partial thromboplastin and thrombin times. Further analysis demonstrated normal levels of von Willebrand factor. Platelet aggregation analyses showed decreased response to ristocetin, ADP, and adrenalin, but a normal response to collagen. Platelet transmission electron microscopy (PTEM) showed normal alpha and dense granules (Figure 7; White, 2008). Platelet secretory function was not analyzed due to the unavailability of the test. The platelet aggregation and electron microscopy results suggested platelet dysfunction associated with an intrinsic platelet pathway (or platelet storage pool defect). Epistaxis was controlled by oral tranexamic acid. DDAVP was administered during dental procedures with no requirement for platelet transfusion.

## Discussion

We identified six novel mutations spread throughout the entire coding sequence of *SOX10* in seven patients with WS2, WS4, and PCW/PCWH. The pathogenicity of the mutations was supported by their *de novo* occurrence and evidence from *in vitro* analysis and molecular models. The results suggested that the phenotype could not be predicted based on the genotype alone, and that additional information from *in vitro* studies was essential for making genotype–phenotype predictions. Notably, we observed a higher frequency (80%, 4/5) of WS4-neurological variants among individuals with WS4 in this cohort, compared with a recent review (19%, 15/79) (Song et al., 2016). This apparent discrepancy may have been because of the small sample size of our cohort, and unintentional recruitment bias because less-severe cases were not referred to clinical genetics service.

*SOX10*, located on 22q13.1, contains five exons, including a start codon in exon 3 and the last codon in exon 5. The gene encodes a 466-aa peptide belonging to the SOX family of transcription factors. SOX10 protein possesses an N-terminal dimerization domain (DIM), a high-mobility group (HMG) domain functioning as a DNA-binding site, a SOX E-specific (or K2) domain having context-dependent transactivation activity, and the distal transactivation (TA) domain. SOX10 plays critical roles in the early development of neural crest cells, which contribute to parts of the peripheral and central nervous systems, enteric ganglia, and melanocytes. SOX10 protein, in synergy with PAX3, also strongly transactivates *MITF* expression (Bondurand et al., 2000, 2007; Kelsh, 2006; Cossais et al., 2010). A total of 181 *SOX10* mutations have been described in patients with WS and/or Kallmann syndrome (KS), of which 55% are truncating mutations (nonsense, frameshift, and splicing defect) and null alleles (gross rearrangement or deletion), while the remainder represent missense variants. Previous studies indicated that more severe phenotypes, PCWH, were associated with truncating mutations in the last coding exon (exon 5), because the resulting mRNAs escaped NMD, leading to production of the mutant protein and triggering of a potent dominant-negative process (Pingault et al., 1998; Inoue et al., 2004; Bondurand et al., 2007). In contrast, truncating variants with mutations in the first two coding exons (exons 3 and 4) were considered to activate the NMD machinery, resulting in less-severe phenotypes (Inoue et al., 2004). Missense mutations were generally thought to cause more-severe manifestations via the dominant-negative mechanism. Information on the functional characterization of missense *SOX10* variants is limited, however, additional data on *SOX10* mutations and their associated phenotypes (Cossais et al., 2010; Chaoui et al., 2011; Zhang et al., 2012; Fernandez et al., 2014) have highlighted the fact that phenotypes cannot be predicted accurately based on the genotype alone, thus warranting further investigation.

Several possible mechanisms could account for the underlying platelet dysfunction in the current patients with p.Phe392Cysfs^∗^117 variant, including a coincidental bleeding diathesis disorder, digenic inheritance between *SOX10* and genes regulating platelet functions, shared sequence homology between the additional 116 aa and platelet function-related protein(s), and a unique effect of the elongation segment via *SOX10* up-regulating genes involved in platelet function or by other, yet unknown mechanism. We excluded the first two possibilities because of the absence of candidate variants in the target genes analyzed, including those underlying pigmentation disorders with platelet storage pool defects, such as Hermansky–Pudlak syndrome and non-syndromic oculocutaneous albinism (OCA) with platelet dysfunction (caused by biallelic mutations in *TYR* and *TYRP*). In addition, our patients had normal numbers of alpha and dense granules per platelet, thus excluding Hermansky–Pudlak syndrome (absent/decreased dense granules) and non-syndromic OCA associated with platelet dysfunction (decreased dense granules) (Power et al., 2019). We investigated the third hypothesis of shared sequence homology by BLAST analysis of 116-aa SOX10-unrelated proteins and found no matching proteins, thus excluding this option.

A recent study showed that SOX10 may play roles in activating several previously undescribed genes, including genes involved in integrin and focal adhesion kinase (FAK) signaling (Fufa et al., 2015). Biallelic mutations of integrin αIIbβ3 are known to cause Glanzmann thrombasthenia, a platelet disorder characterized by lack of platelet aggregation to all stimuli (Nurden, 2006). FAK and Pyk2 belong to the focal adhesion kinase family and are both involved in platelet biogenesis and activation (Guidetti et al., 2019). Mutations of these two genes have not been associated with any human disorders to date, and no pathogenic variants of αIIbβ3, FAK, or Pyk2 were found in our patients with platelet dysfunction. In summary, we failed to identify the mechanism underlying the platelet disorders in our patients, but suggest that they were caused by a specific insult of the frameshift and the elongated segment of the p.Phe392Cysfs^∗^117 protein variant. Inoue et al., have shown that molecular pathogenesis of extension mutations of *SOX10* for PCWH is different from that caused by premature truncation mutations and that the deleterious effect of the extension tail may act through a gain-of-function mechanism (Inoue et al., 2007). The extension tail could diminish transcriptional activity and DNA-binding and transactivation activity (Inoue et al., 2007). Such examples could support the toxic effect of the elongation tail in our twin patients with platelet dysfunction.

Pathogenic variants of *SOX10* account for 30% of cases of KS (hypogonadotropic hypogonadism and anosmia due to olfactory bulb agenesis) with deafness (Pingault et al., 2013). We therefore searched literature and public databases for reports of specific mutation(s) resulting in both disorders, and identified p.Ser30^∗^ as a novel variant recently reported in a sporadic KS individual with unknown hearing status and inheritance (Cassatella et al., 2018). The p.Trp142Ser mutation detected in our patient has not been described in KS, however, distinct mutations of this codon have been noted, including Trp142Arg in a KS individual with deafness (Pingault et al., 2013), p.Trp142Cys in WS2 with heterochromia only (Ideura et al., 2019), and Trp142^∗^ (ClinVar VCV000505252) in a patient with genetic deafness (Landrum et al., 2018). There has been no report to date of both WS and KS occurring in the same family, with a shared mutation, suggesting that the genetic background or modifier genes shared by family members may play critical roles in determining the final phenotype. *In vitro* studies of each variant are necessary to provide a better understanding of the pathogenic mechanisms and better phenotype predictions. Additional *in vivo* analysis would further improve our understanding of the phenotypic effects of *SOX10* variants, such as why patients develop WS and not KS, and vice versa.

The present results also suggested that not only the location (first two coding vs. last coding exons) and type (missense vs. truncation/frameshift) of *SOX10* mutations, but also the altered levels of protein expression of the *SOX10* missense variants and their molecular weight, including the aa composition (ratio of charged/hydrophobic aas) of the mutant proteins, may determine the severity of the expressed phenotype (Junod et al., 2020). These results also suggest that phenotypic severity may be regulated by the differential expression of each mutant protein, influenced by the activation versus escaping NMD pathway and other yet unknown factors. Other studies have noted conflicting data on phenotype–genotype correlations based on predictions using genotype alone (Bondurand et al., 2007; Chaoui et al., 2011; Zhang et al., 2012).

This study had some limitations, including its small sample size. In addition, some mutations were not detectable by the current methods, such as exon-level deletion/duplications, gross gene rearrangements, and variants in promoter or deep intronic sequences. Furthermore, we did not perform co-expression or competition assays to confirm the dominant negative effects or haploinsufficiency of the mutant *SOX10* genes.

In conclusion, genotype–phenotype correlations in patients with *SOX10*-related WS are affected by the site and type of the mutation, the molecular weight and aa content of the resulting protein, which may affect its nuclear transport, the level of protein expression, and the genetic background (modifier genes) of the individuals. We suggest that *in vitro* and *in vivo* analysis of *SOX10* mutations is required to further our understanding of the mechanisms resulting in specific WS subtypes, and to allow better predictions of genotype–phenotype correlations.

## Data Availability Statement

The datasets presented in this study can be found in online repositories. The names of the repository/repositories and accession number(s) can be found in the article/Supplementary Material.

## Ethics Statement

The studies involving human participants were reviewed and approved by the Ramathibodi Hospital Human Research Ethics Committee (MURA2020/577 and MURA2020/884). Written informed consent to participate in this study was provided by the participants’ legal guardian/next of kin.

## Author Contributions

ST performed the molecular and functional experiments and prepared the manuscript draft. NJ designed and supervised the functional study and performed cytogenomic microarray analysis. AJ performed the bioinformatics analysis. LTJ performed the molecular modeling analysis and wrote part of the manuscript. IC, KR, TT-A, and KL collected and interpreted the clinical data. MS-S assisted with the functional study. NS analyzed the hematologic data. DW obtained the research grants, designed the study concept, and prepared and revised the manuscript. All authors reviewed and approved the final manuscript.

## Conflict of Interest

The authors declare that the research was conducted in the absence of any commercial or financial relationships that could be construed as a potential conflict of interest.

## Footnotes

1 http://frodo.wi.mit.edu

2 https://sift.bii.a-star.edu.sg/

3 http://genetics.bwh.harvard.edu/pph2/

4 www.fruitfly.org/seq_tools/splice.html

5 https://www.ncbi.nlm.nih.gov/clinvar

6 http://www.biobase-international.com

7 https://varsome.com/variant/hg19

8 https://www.broadinstitute.org/
