# Supplementary material for: Novel SOX10 Mutations in Waardenburg Syndrome: Functional Characterization and Genotype-Phenotype Analysis
Source: Front Genet. 2020 Dec 9;11:589784. doi: 10.3389/fgene.2020.589784 (PMC7756068; doi:10.3389/fgene.2020.589784)
Supplement: Supplementary file 1 [file Data_Sheet_1.pdf]

frontiers

Supplementary Material

Novel *SOX10* Mutations in Waardenburg Syndrome: Functional Characterization and Genotype-Phenotype Analysis. Thongpradit et al.

Table S1 Primer sequences and PCR conditions for *MITF*-isoform A

Exon	Sequence (5′-3′)	Annealing temperature (°c) [a]
1F	TGGGAGCTGTAGTTTTCGTG	63
1R	CTCTCCTCGCCCCAGAGT	
2F	TGGAGTACCATTCTGTGACTTGA	60
2R	ACACCTAGCAAATGGAAAATGG	
3F	TCATGTTTGTGCCTGAAGGA	60
3R	CTTTTCCACCTCCCCTTCTC	
4F	CTGTGCCATCAGCTTTGTGT	60
4R	TGCTTAAGTTTTCAGGAAGGTG	
5F	CCAATGCTTGGCAACTCATA	60
5R	GAACCCTGGAAACACCTCAA	
6F	CAAAGGGAACTGGTTGAGGA	60
6R	TGTTTTAACCACTGCAGAGACC	
7F	GCTTTTGAAAACATGCAAGC	60
7R	CAGCTGTAGGAATCAACTCTCC	
8F	CGTTGTCATGACCTGGAGAA	60
8R	CCTTTTGCACAATTCAATGC	
9F	GTACACGGCTTGGGTGGT	60
9R	GTCAACTCCCCTATGGCTCA	
10pF	CTAATGACGCGCATCTACCA	60
10pR	TCCTGGGCTATTGATAAAGCA	
10dF	AACTGCTTCCTTTCTTGATTCG	60
10dR	CCCTTCAGTTTCGGTTGGTA	

[a] PCR condition: initial denaturation at 94°C 5 min; 35 cycles of denaturation at 94°C for 45 sec, annealing for 45 sec (but for exon1 annealed at 63°C), extension at 72°C for 45 sec; and final extension at 72°C for 7 min. Reference sequences of MITF: NT_022517.19, NM_198159.2, NP_937802.1

Table S2 SOX10 primers and sequences for PCR-sequencing[a]

Primer name	Sequence (5´-3´)	Annealing temp (°C)	Product size (bp)
SOX10 E1F	GAGTGTTGGGGATGAAGGAA	63	447
SOX10 E1R	AACGGGGTTTAGAGGAGAGC		
SOX10 E2F	GGTTTAGCTGGAGCAGGAAG	58.5	724
SOX10 E2R	GACAGTCCCGCTCTGAGGT		
SOX10 E3F	TCCAAGATGGACACTCAGAGG	58.5	450
SOX10 E3R	AGAGTCCAGGGTCTCATTGC		
SOX10 E4F	GTGAACCATGGAAGTTCACG	58.5	916
SOX10 E4R	GACCTGTCAGCCTCTTCAGC		

[a] PCR condition: initial denaturation at 94°C 5 min; 35 cycles of denaturation at 94°C for 45 sec, annealing for 45 sec, extension at 72°C for 45 sec (but 2 min for exon 1); and final extension at 72°C for 7 min. Reference sequences of SOX10: NT_011520.11, NM_006941.3, NP_008872.1

Table S3 SOX10 mutagenesis primers[a]

Primer Name	Primer Sequence (5´-3´)
SRY-bX30F	GGGAGCGCGCCCTAGCTAGGGCCCGAC
SRY-bX30R	GTCGGGCCCTAGCTAGGGCGCGCTCCC
SRY-bX142F	GCTGGGCAAGCTCTCGAGGCTGCTGAACGAAAGTG
SRY-bX142R	CACTTTCGTTCAGCAGCCTCGAGAGCTTGCCCAGC
SRY-bX160F	GAGGCTGAGCGGCCCCGTATGCAGCAC
SRY-bX160R	GTGCTGCATACGGGGCCGCTCAGCCTC
SRY-bX71F	CGATGACAAGTTCCCTGTGCATCCGCGAG
SRY-bX71R	CTCGCGGATGCACAGGGAACTTGTCATCG

[a] PCR condition for site-directed mutagenesis: initial denaturation at 95°C for 1 min; 18 cycles of denaturation at 95°C for 50 sec, annealing at 60°C for 50 sec, extension at 68°C for 9 min; followed by final extension at 68°C for 7 min.

Table S4 Subcellular localization of different SOX10 protein variants

SOX10 variant	Total cells	Subcellular localization (%)		
		nuclear	cytosolic	nucleocytosolic
Wild type	151	124 (82)	7 (5)	20 (13)
p.Trp142Ser	158	131 (83)	0 (0)	27 (17)
p.Leu160Pro	185	158 (85)	4 (2)	23 (13)
p.Cys71Hisfs*62	132	0 (0)	132 (100)	0 (0)
p.Ser30*	121	5 (4)	113 (93)	(3)

1.1 Supplementary Figures

SOX10

Lane	Band labeled	Relative front	Adjusted volume (Int)	Volume (Int)	SOX10: relative quantity to WT	Adjusted relative quantity to WT[a]	% change of protein synthesized, compared to WT	Level of protein synthesis relative to WT
1	WT	0.322034	8253319	9658455	1	1		
2	Trp142Ser	0.322034	2138020	2968901	0.259	0.296	70% reduction	30% of NL
3	Leu160Pro	0.313559	983309	1650155	0.119	0.111	89% reduction	11% of NL
4	Cys71Hisfs*62	0.754237	42910973	44148258	5.199	4.956	500% increase	500% of NL
5	Ser30*	NA			NA	NA	NA	NA

[a] adjusted relative quantity of each SOX10 variant according to the GAPDH on the individual lane was achieved by applying the rule of three in arithmetic

GAPDH

Lane	Band labeled	Relative front	Adjusted volume (Int)	Volume (Int)	GAPDH: relative quantity to WT
1	WT	0.45339	3307041	4126050	1
2	Trp142Ser	0.449153	2892475	3677140	0.875
3	Leu160Pro	0.444915	3555240	3962015	1.075
4	Cys71Hisfs*62	0.440678	3468267	3915534	1.049
5	Ser30*	0.440678	2863007	3255896	0.866

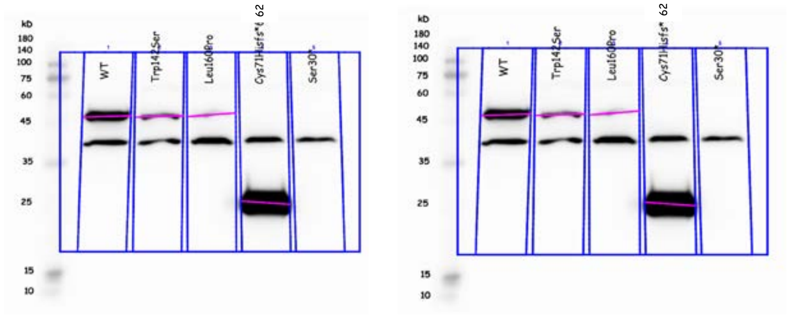

Figure S1. Measuring level of wildtype and mutant SOX10 protein synthesis

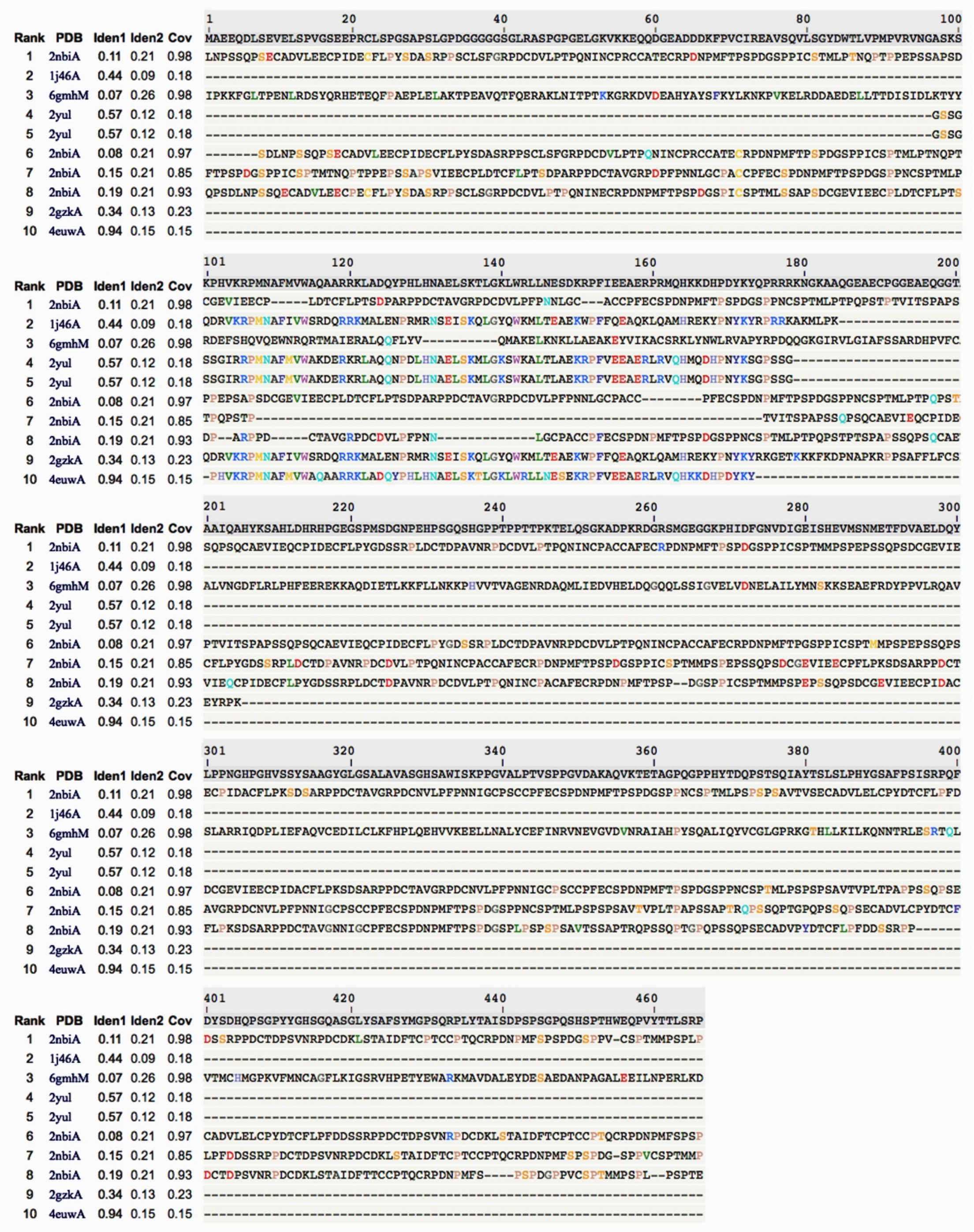

Figure S2 Alignment and identity of templates used in molecular modeling of Sox10. Sequence highlighted in color are identical to the corresponding residues in Sox10. The coloring indicates chemical properties of amino acids: polar residues are brightly colored and non-polar residues dark shade. “Ident1” is the percentage sequence identity of the templates in the threading aligned region with the query sequence. “Ident2” is the percentage sequence identity of the whole template chains with query sequence. “Cov” represents the coverage of the threading alignment and is equal to the number of aligned residues divided by the length of query protein.
